# lncRNA-FMR6 directly binds SAV1 to increase apoptosis of granulosa cells in premature ovarian failure

**DOI:** 10.1186/s13048-023-01121-5

**Published:** 2023-04-01

**Authors:** Dongqin Bao, Lei Gao, Haiyan Xin, Lie Wang

**Affiliations:** 1 Center for Reproductive Medicine, The Affiliated Shuyang Hospital of Xuzhou Medical University, Suqian City, Jiangsu Province 221004 China; 2 Reproductive Medicine Center of Qingdao Women and Children’s Hospital, Qingdao City, Shandong Province 266034 China

**Keywords:** lncRNA-FMR6, SAV1, Premature ovarian failure, Granulosa cells

## Abstract

**Background:**

A regulatory mechanism of lncRNA binding to protein has been detected in premature ovarian failure (POF). Therefore, this study was expected to illustrate the mechanism of lncRNA-FMR6 and SAV1 regulating POF.

**Methods:**

Follicular fluid and ovarian granulosa cells (OGCs) from POF patients and healthy volunteers were collected. Using RT-qPCR and western blotting, lncRNA-FMR6 and SAV1 expression were detected. KGN cells were cultured, and the subcellular localization analysis of lncRNA-FMR6 was carried out. In addition, KGN cells were treated with lncRNA-FMR6 knockdown/overexpression or SAV1 knockdown. Then, cell optical density (proliferation), apoptosis rate, Bax and Bcl-2 mRNA expression were explored by CCK-8, caspase-3 activity, flow cytometry and RT-qPCR analysis. By performing RIP and RNA pull-down experiments, the interactions among lncRNA-FMR6 and SAV1 was investigated.

**Results:**

Up-regulation of lncRNA-FMR6 was shown in follicular fluid and OGCs of POF patients, and ectopic overexpression of lncRNA-FMR6 promoted KGN cells apoptosis and inhibited proliferation. lncRNA-FMR6 was localized in the cytoplasm of KGN cells. SAV1 bounding to lncRNA-FMR6 was negatively regulated by lncRNA-FMR6, and was down-regulated in POF. SAV1 knockdown promoted KGN cells proliferation and inhibited apoptosis, and partially eliminated the effect of lncRNA-FMR6 low expression on KGN cells.

**Conclusion:**

Overall, lncRNA-FMR6 accelerates POF progression by binding to SAV1.

## Introduction

Premature ovarian failure (POF) is a rare clinical disease which poses serious threat to women’s reproductive health, with the prevalence of approximately 1% across the world [1]. POF has been reported to be characterized by amenorrhea, elevated gonadotropins and estrogen deficiency, with patients’ ovarian function ceasing before the age of 40 years [2]. At present, hormone replacement therapy is the most commonly adopted for POF treatment, while, it may also cause a series of side effects including depression and vaginal bleeding [3, 4]. Hence, there is an urgent need to develop new POF therapies. According to a previous research, POF results in a decrease in the number of follicles, the state and the density of ovarian granulosa cells (OGCs) [5]. Additionally, genetic causes, such as type 1A pseudohypoparathyroidism (GNAS1 gene), fragile X syndrome (FXS, FMR1 gene), or Turner syndrome, have been reported to be show association with POF [6]. Therefore, focusing on the pathological alterations and genetic abnormalities in OGCs may provide novel insights into POF treatment.

Long non-coding RNAs (lncRNAs) are non-protein-coding RNAs over 200 nucleotides in length regulating gene expression at multiple levels [7]. LncRNA is a crucial regulatory molecule in the occurrence of various human diseases, including reproduction, and is a key regulator for the normal development of OGCs, follicles and ovaries, and can play the role of a biomarker for OGCs related diseases [8]. For example, lncRNA BANCR is upregulated in OGCs from patients undergoing polycystic ovary syndrome, and its overexpression inhibits proliferation and promotes apoptosis in KGN cells (human ovarian granulosa cells, GCs) [9]. LncRNA MALAT1 is downregulated in OGCs from patients suffering from endometriosis, and its knockdown inhibits proliferation of OGCs [10]. lnc-HSPA6–2, a risk factor-related lncRNA for ovarian hyperstimulation syndrome, was significantly down-regulated in OGCs [11]. In addition, multiple lncRNAs, including HOTAIR [12], NEAT1 [13] and DLEU1 [14] are risk warning factors for POF and can promote apoptosis of GCs. LncRNA-FMR6 is a splice antisense-oriented lncRNA in FXS that overlaps with exon 15–17 of Fragile X Mental Retardation 1 (FMR1) and participates in the regulation of FMR1 gene transcription [15]. The involvement of lncRNA-FMR6 in the development of fragile X-associated premature ovarian failure (FXPOI) has been reported, with a negative linear correlation between lncRNA-FMR6 expression in GCs and the number of oocyte retrieved [16]. Nevertheless, the exact mechanism of lncRNA-FMR6 in POF development still remains to be identified.

Salvador family WW domain containing protein 1 (SAV1), a WW domain-containing protein that is a core kinase component of the Hippo signaling pathway, is expressed in mammalian ovaries and exerts a vital role in controlling follicle development [17–19]. Previous studies have revealed that SAV1 exerts an inhibitory role in ovarian follicular development by regulating the proliferation of granulosa cells in graded pre-follicles (6–8 mm in diameter) [20]. Studies have demonstrated that lncRNA exerts its biological functions mainly through interaction with RNA-binding proteins [21]. Moreover, reports suggest that SAV1 can directly bind to lncRNA and participate in regulating the biological function of cells [22]. However, there are few reports on the mechanism by which lncRNA, especially lncRNA-FMR6, regulates SAV1 in POF.

In the present research, we examined the differential expression of lncrNA-FMR6 in POF and explored its regulatory relationship with SAV1. We hypothesized that lncRNA-FMR6 promoted apoptosis of GCs cells in POF by binding to SAV1 and negatively regulating SAV1 expression. The findings of this project are expected to develop new and valuable molecular targets for the clinical treatment of POF.

## Methods

### Patients

We recruited a total of 24 patients with POF who received IVF/ICSI-ET and 24 controls. After transvaginal ultrasound oocytes were collected from the patient, blood free follicular fluid was removed from large follicles larger than 14 mm. Moreover, this study was approved by the Accreditation Committee of Reproductive Medicine Institutions of our hospital and informed consent was obtained from all the participants. The inclusion criteria of patients with POF included: basal serum FSH ≥10 IU/l, age < 40 years, menstrual cycle 23–35 days, and unilateral ovarian AFC < 5. Women with a history of chemotherapy, radiation or ovarian surgery were excluded. Besides, women with infertility due to male factors or fallopian tube obstruction, normal FSH (< 10 IU/l), and normal menstrual cycle served as controls. Table 1 summarizes the clinical characteristics of all the participants.

**Table 1:** Baseline data of both patients and controls

Variables	Control (***n*** = 24)	POF (***n*** = 24)
Age (year)	29.37 ± 3.46	30.15 ± 4.20
BMI (kg/m^2^)	21.96 (19.84, 22.66)	22.01 (19.19, 25.37)
AMH (ng/mL)	3.25 (2.20, 5.09)	0.42 (0.26, 0.74) **
basal LH (IU/L)	5.33 (3.72, 7.85)	5.49 (3.96, 8.47)
basal E2 (pg/mL)	29.98 (23.14, 43.61)	28.44 (13.65, 41.83)
basal FSH (IU/L)	6.08 (4.77, 7.05)	13.48 (11.71, 21.32) **

** *P*-value < 0.05 vs. Control

### Cell culture

On the day of oocyte extraction, follicular fluid (> 14 mm, the follicle size was observed dynamically under the monitoring of B ultrasound, and the path line was measured) from each participant’s large follicle was collected. By discarding supernatant after centrifugation, the precipitate was incubated with hyaluronidase (80 IU/ml) (Sigma, USA) at 37 °C for 30 min. Later, it was centrifuged and transferred to a lymphocyte medium (Solarbio, China). The OGCs were isolated from the interlayer phase, and injected into PBS. In the end, the separated OGCs were flash-frozen and preserved at − 80 °C.

To detect gene expression in OGCs of POF patients, OGCs of POF patients and control volunteers were cultured. In addition, KGN cells were obtained from RIKEN Biological Resource Center (Japan), and cultured to detect gene regulation on KGN cells in vitro. OGCs and KGN cells were re-suspended in 10% FBS added DMEM/F12 medium (Gibco), inoculated in 48-well plates at the rate of 5 × 10^4^ cells/well, and also placed in an incubator containing including 5% CO_2_ at 37 °C for conventional culture.

### RT-qPCR

RNAiso Plus (Takara, Japan) was adopted in the extraction of total RNA, and PrimeScript RT Kit (Takara) was employed to generate the cDNA (2 μg of total RNA). Subsequently, PCR was performed with the use of Hieff qPCR SYBR Green Master Mix kit (Yeasen, China) in an ABI 7500 Real-Time PCR System (Applied Biosystems). lncRNA-FMR6 and SAV1 expression were determined using the delta-delta Ct (2^-∆∆Ct^) method, and normalized to GAPDH. Table 2 lists all the used primers.

**Table 2:** Primer sequences used for real-time PCR analysis

Genes	Primers (5′-3′)
lncRNA-FMR6	Forward: AGCACTTCAGGGCAGATTTT
	Reverse: TGGTGAATGATCACCCAATG
SAV1	Forward: ATGAGGCGTGAAAGCAACAG
	Reverse: CCGCTGTGCTCATAGTATCTGTA
GAPDH	Forward: GTCAACGGATTTGGTCTGTATT
	Reverse: AGTCTTCTGGGTGGCAGTGAT

### Subcellular localization

To investigate whether lncRNA-FMR6 performs its function in the cytoplasm, subcellular localization analysis was performed. This assay was conducted with the application of PARIS Kit (Invitrogen). In brief, cytoplasm and nuclear components were obtained from KGN cells by cell fractionation buffer. Next, the nucleus was lysed by the cell disruption buffer. To evaluate the relative expressions of lncRNA-FMR6, RT-qPCR was carried out, U6 and GAPDH were employed used as control of nucleus and cytoplasm, respectively.

### Cell transfection

The siRNA against lncRNA-FMR6, SAV1 or negative control (si-FMR6, si-SAV1 and si-NC; 50 nM; Gene-Pharma, USA) were transfected into KGN cells (1 × 10^5^ cells/well in 48 well plate) using Lipofectamine 2000 (Invitrogen, USA). Additionally, lncRNA-FMR6 overexpression vector (FMR6-pcDNA; 2 μg/mL) and empty vector (2 μg/mL) were acquired from GenePharma (China). 48 h post-transfection, cells were collected, and RT-qPCR was performed to assess the efficiency of the transfections.

### CCK-8 assay

The CCK-8 Kit (Cat#: C0038; Beyotime, China) was adopted for assessing the proliferation of cells in the study. Briefly, KGN cells (1 × 10^4^) were injected into 96-well culture plates. After 24, 48 and 72 h of culturing, 10 μL of CCK-8 solution was poured in all wells and incubated at 37 °C for 2 hours in the dark. Microplate spectrophotometer (Bio-Rad Laboratories, USA) was employed to measure the value of each well in these plates, which were subjected to the wavelength of the radiation of 450 nm.

### Caspase-3 activity assay

Caspase-3 activity was measured using the caspase-3 colorimetric assay kit (Ca# ab39401, Abcam, UK). The KGN cells were collected, lysed with lysate and centrifuged at 10000 g for 5 min at 4 °C. The protein concentration of the precipitate was lysed with the use of a BCA Kit (Pierce, USA). The protein concentration was adjusted to 2 μg/μL Cell Lysis Buffer. Then, 50 μL of reaction buffer containing 10 nM DTT and 5 μ L of DEVD-ρNA substrate were supplemented to the lysate sample. After the incubation at 37 °C for 2 hours, caspase-3 activity was identified at 405 nm under a microplate spectrophotometer.

### Apoptosis assay

Annexin V-FITC- PI (BestBio, China) was carried out to measure cell apoptosis. Briefly, transfected KGN cells were seeded in 24 well plates at 5 × 10^4^ cells/ml (1 ml/well). Following 48 h incubation, cells were harvested and washed with PBS. Next, Annexin V-FITC and PI staining reagents were added to incubate for 15 min at room temperature in the dark. In addition, cell apoptosis rate was evaluated through FACScan flow cytometer (BD Biosciences, USA).

### Western blotting

Total proteins were obtained from KGN cells lysed with a RIPA buffer (Beyotime) for 30 min. Later, the protein concentration was determined using a BCA Kit, and 10% SDS-PAGE was adopted for separating 20 μg of the protein samples before their transfer onto a PVDF membrane (Millipore). Subsequently, the PVDFs were sealed in 5% skimmed milk at 37 °C for 60 min, and incubated at 4 °C for 12 h with SAV1 (Ca# ab172705, 1:1000, Abcam, UK) and GAPDH (Ca# ab8245, 1:2000, Abcam) antibodies. This was also followed by an hour of incubation at 37 °C with a suitable second antibody (Ca# ab205718, 1:5000, Abcam). Finally, blots were visualized based on an Immobilon ECL substrate (Millipore). Bio-Rad’s Image Lab Software was used to scrutinize the gray of proteins.

### RNA pull-down assay

RNA pull-down assay was conducted to identify the interaction between lncRNA-FMR6 and SAV1 protein. In brief, the DNA probe complementary to lncRNA-FMR6 was synthesized and biotinylated by GenePharma Co., Ltd. (Shanghai, China). Following the instruction of the manufactures, a Magnetic RNA-Protein Pull-Down Kit (Thermo Fisher) was adopted for completing the RNA pull-down assay. The RNA-binding protein complexes were washed and eluted for performing western blot analysis.

### RNA immunoprecipitation (RIP)

To evaluate the interaction between lncRNA-FMR6 and SAV1, the current experiment was performed utilizing RNA-binding protein immunoprecipitation kit (Millipore, USA). Briefly, magnetic beads were incubated with SAV1 (Abcam) or IgG antibody (Millipore) vortically at 25 °C for 30 min. KGN cells at 80% confluency were treated with RIP lysis buffer. In addition, the cell lysis solutions (100 μl) were subject to pretreated magnetic beads at 4 °C overnight. Then, beads were washed, and SAV1 protein levels were detected via western blot. Meanwhile, lncRNA-FMR6 enrichment level was quantified by RT-qPCR.

### Statical analysis

GraphPad Prism 7.0 (GraphPad Software, USA) was utilized for the statistical analysis of the data in the format of mean ± SD from three independent experiments. Pearson’s correlation coefficient was utilized to evaluate the associations of lncRNA-FMR6 and SAV1. Unpaired student’s t-test was employed in assessing the difference between the two independent groups. Meanwhile, one-way ANOVA with Tukey’s test was utilized to evaluate the differences among multiple groups. A *P*-value < 0.05 indicates statistical significance.

## Results

### LncRNA-FMR6 overexpressed in POF

**Fig. 1:**
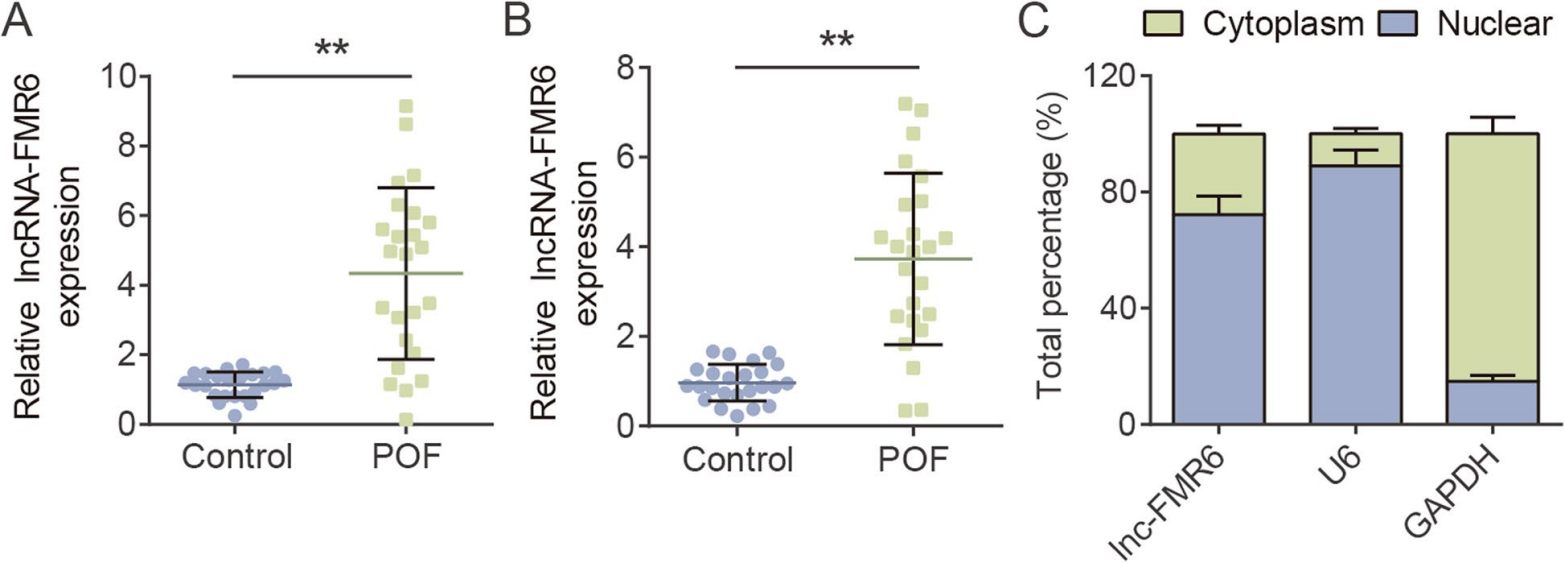
LncRNA-FMR6 overexpressed in POF. **A** Relative levels of lncRNA-FMR6 in follicular fluid of POF patients (*N* = 24) and controls (*N* = 24) were assessed via RT-qPCR. **P < 0.01. **B** Relative levels of lncRNA-FMR6 in OGCs of POF patients (*N* = 24) and controls (*N* = 24) were assessed via RT-qPCR. **P < 0.01. **C** lncRNA-FMR6 transcript abundance in cytoplasm and nucleus of KGN cells, as detected via subcellular fractionation

At first, we identified the differential expression of lncRNA-FMR6 in POF. Follicular fluid and OGCs of 24 patients with POF and 24 controls were collected and explored by RT-qPCR. LncRNA-FMR6 levels in the follicular fluid of POF patients was around 4.5-fold higher than that of the control group (Fig. 1A). Similarly, the expression of lncRNA-FMR6 in OGCs was elevated by approximately 3.6 times (Fig. 1B). In addition, analysis of subcellular localization of lncRNA-FMR6 in KGN cells revealed that lncRNA-FMR6 levels in cytoplasm was higher than that in nucleus (Fig. 1C). lncRNA-FMR6 was mainly located in cytoplasm. Generally, over-expression of lncRNA-FMR6 in POF may regulate the biological function of cells after the transcription.

### LncRNA-FMR6 silencing facilitated KGN cells proliferation and restrained apoptosis, while it upregulation resulted an opposite trend

**Fig. 2:**
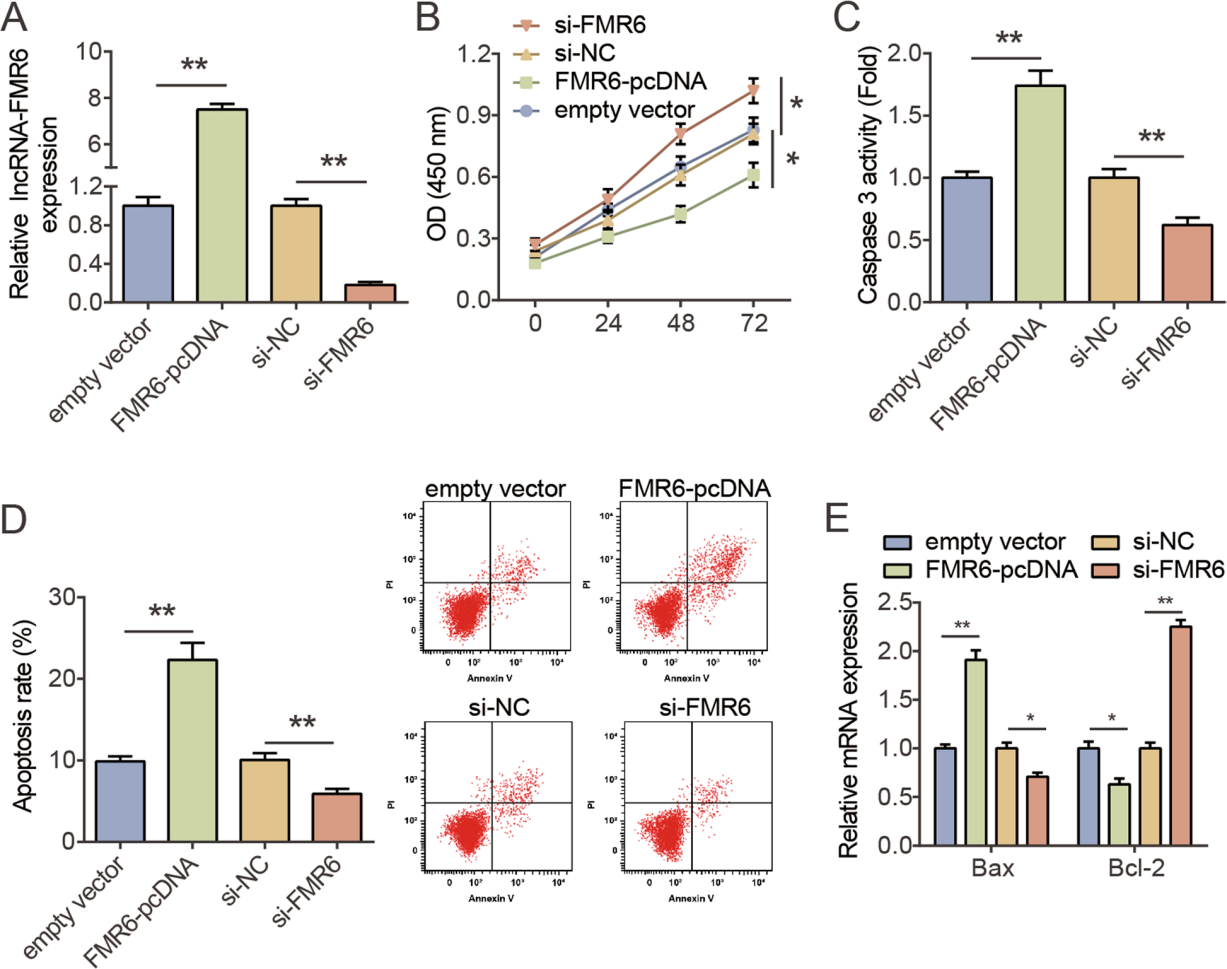
LncRNA-FMR6 silencing facilitated KGN cells proliferation and restrained apoptosis, while it upregulation resulted an opposite trend. **A** Relative levels of lncRNA-FMR6 in KGN cells transfected with either FMR6-pcDNA, empty vector, si-NC and si-FMR6, as assessed via RT-qPCR. **P < 0.01. **B** Effect of FMR6-pcDNA or si-FMR6 transfection on the proliferative ability of cells, as determined through CCK-8. **P < 0.01. **C** Effect of FMR6-pcDNA or si-FMR6 transfection on caspase-3, as determined by flow cytometry assay. **P < 0.01. **D** Effect of FMR6-pcDNA or si-FMR6 transfection on apoptosis rate, as determined by caspase-3 activity assay. **P < 0.01. **E** FMR6-pcDNA or si-FMR6 transfection’s effect on Bax and Bcl-2 mRNA levels, as ascertained via RT-qPCR. **P < 0.01

To evaluate the effect of lncRNA-FMR6 on GCs proliferation and apoptosis in vitro, KGN cells were treated with lncRNA-FMR6 overexpression or knock-down. RT-qPCR analysis demonstrated that lncRNA-FMR6 levels in FMR6-pcDNA group were increased by around 7.5-fold compared with empty vector, and the level of lncRNA-FMR6 in si-FMR6 group was decreased by approximately 80% compared with si-NC (Fig. 2A). Next, we evaluated the biological activity of lncRNA-FMR6 in KGN cells. As displayed in Fig. 2B, CCK-8 manifested KGN cells proliferation was obviously suppressed after over-expression of lncRNA-FMR6, while was promoted after the low expression of lncRNA-FMR6 (Fig. 2B). Additionally, the Caspase-3 activity of KGN cells transfected with FMR6-pcDNA vector was higher than that of transfected with FMR6-pcDNA vector, while transfected with si-FMR6 group was lower than that of si-NC (Fig. 2C). Similarly, flow cytometry analysis demonstrated that over-expression of lncRNA-FMR6 promoted KGN cell apoptosis, while lncRNA-FMR6 knockdown inhibited apoptosis (Fig. 2D). In addition, we observed that over-expression of lncRNA-FMR6 resulted in to an amplification in Bax and a reduction in Bcl-2 levels in KGN cells, whereas lncRNA-FMR6 silencing contributed to a decrease in Bax and an increase in Bcl-2 levels (Fig. 2E). The obtained results suggested that lncRNA-FMR6 promoted KGN cells apoptosis and inhibited proliferation.

### SAV1 was low expressed in POF and bound to lncRNA-FMR6

**Fig. 3:**
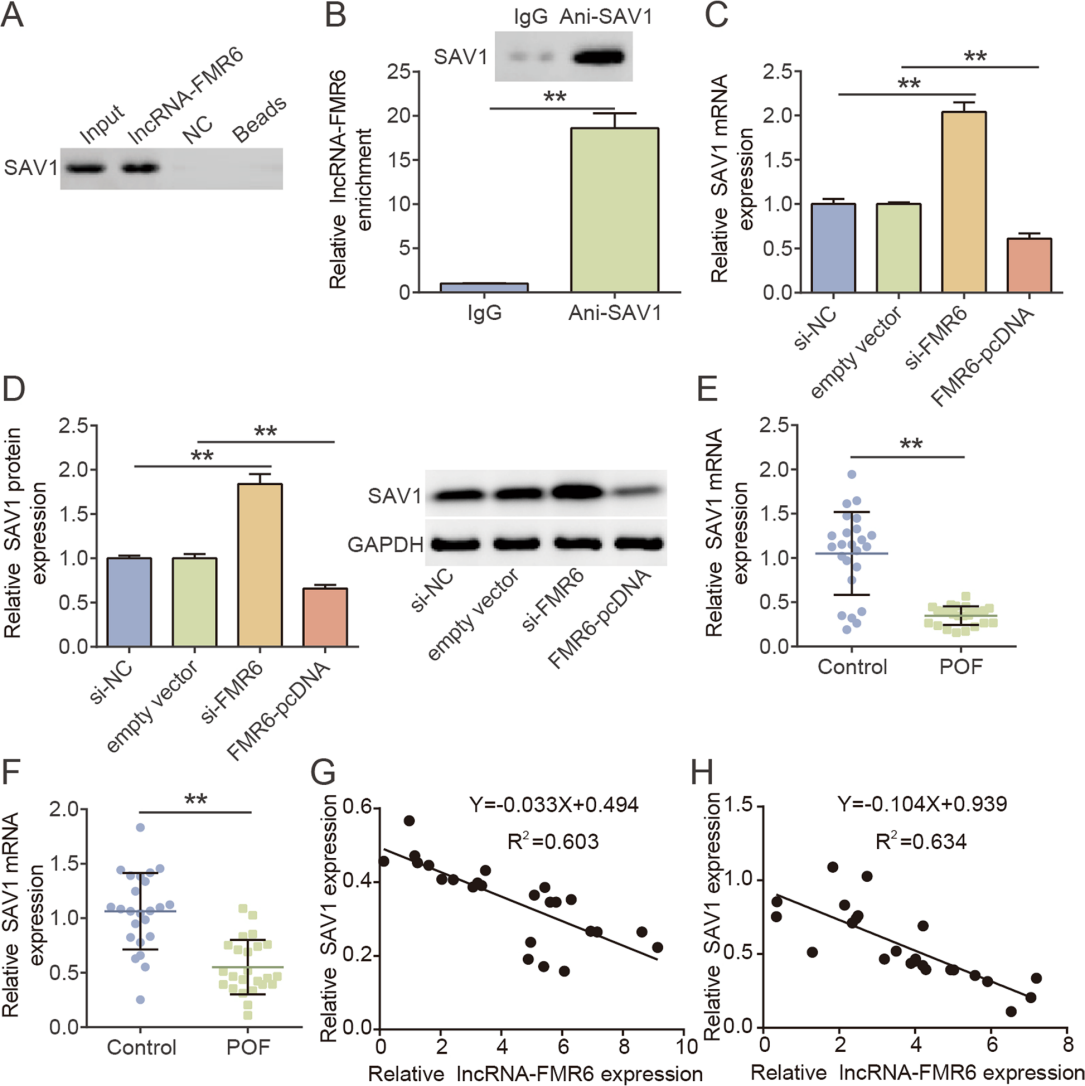
SAV1 was low expressed in POF and bound to lncRNA-FMR6. **A** The interaction among lncRNA-FMR6 and SAV1 was detected via RNA pull-down assay. **B** RIP was performed to evaluate KGN cells extracts with rabbit monoclonal anti-IgG or anti-Ago2. RNA levels in immunoprecipitates were determined by qPCR. **P < 0.01. **C** Relative levels of SAV mRNA in KGN cells transfected with either FMR6-pcDNA, empty vector, si-NC and si-FMR6, as assessed via RT-qPCR. **P < 0.01. **D** SAV1 protein levels in KGN cells transfected with either FMR6-pcDNA, empty vector, si-NC and si-FMR6, as assessed via western blotting. **P < 0.01. **E** Relative levels of SAV1 in follicular fluid of POF patients (*N* = 24) and controls (*N* = 24) were assessed via RT-qPCR. **P < 0.01. **F** Relative levels of SAV1 in OGCs of POF patients (*N* = 24) and controls (*N* = 24) were assessed via RT-qPCR. **P < 0.01. **G** Pearson’s correlation analysis between lncRNA-FMR6 and SAV1 expressions in follicular fluid of POF patients. **H** Pearson’s correlation analysis between lncRNA-FMR6 and SAV1 expressions in OGCs of POF patients

Next, we investigated the binding among lncRNA-FMR6 and SAV1.The expression of SAV1 in lncRNA-FMR6 pull-down complex was measured using western blotting. Data revealed that SAV1 was extensively aggregated in the lncRNA-FMR6 pull-down complex in comparison with NC (Fig. 3A). RIP method was adopted for confirming the combination of lncRNA-FMR6 and SAV1. In relative to IgG, lncRNA-FMR6 were highly expressed in SAV1 antibody (Fig. 3B). The above results demonstrated the combination among lncRNA-FMR6 and SAV1. Subsequently, RT-qPCR and western blotting were utilized to analyze the regulation of lncRNA-FMR6 on SAV1. It was observed that the abnormal expression of lncRNA-FMR6 did influence the expression of SAV1, lncRNA-FMR6 knock-down promoted the level of SAV1 levels and lncRNA-FMR6 overexpression inhibited SAV1 levels (Fig. 3C-D). Furthermore, RT-qPCR revealed that SAV1 mRNA level was down-regulated in follicular fluid and OGCs of patients with POF, and negatively related to lncRNA-FMR6 expression (Fig. 3E-H). Based on the obtained data, it was concluded that the SAV1 bound directly to lncRNA-FMR6 and is negatively regulated by lncRNA-FMR6.

### LncRNA-FMR6-dependent inhibitory effects of SAV1 on KGN cells apoptosis

**Fig. 4:**
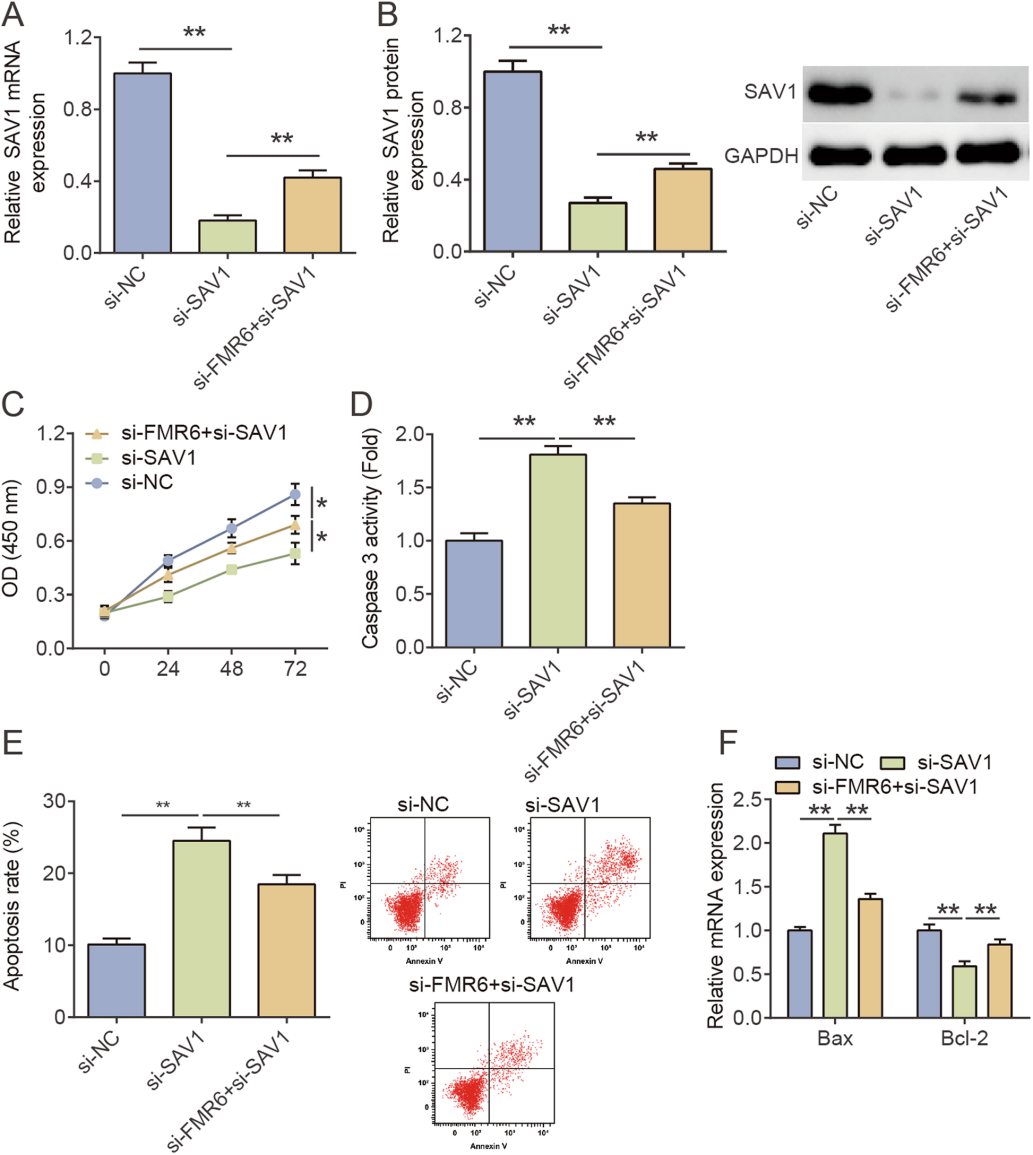
LncRNA-FMR6-dependent inhibitory effects of SAV1 on KGN cells apoptosis. **A** SAV1 mRNA levels in KGN cells delivered with si-NC, si-FMR6 or si-FMR6 + si-SAV1, as assessed via RT-qPCR. **P < 0.01. **B** SAV1 protein levels in KGN cells delivered with si-NC, si-FMR6 or si-FMR6 + si-SAV1, as assessed via western blotting. **P < 0.01. **C** Effect of si-NC, si-FMR6 or si-FMR6 + si-SAV1 transfection on the proliferative ability of cells, as determined by CCK-8 assay. **P < 0.01. **D** Effect of si-NC, si-FMR6 or si-FMR6 + si-SAV1 transfection on caspase-3 activity, as determined by caspase-3 activity assay. **P < 0.01. **E** Effect of si-NC, si-FMR6 or si-FMR6 + si-SAV1 transfection on apoptosis rate, as determined by flow cytometry assay. **P < 0.01. **F** si-NC, si-FMR6 or si-FMR6 + si-SAV1 transfection’s effect on Bax and Bcl-2 mRNA levels, as ascertained via RT-qPCR. **P < 0.01

Subsequently, we verified whether lncRNA-FMR6 promoted KGN cells apoptosis by binding SAV1. As displayed in Fig. 4A-B, si-SAV1 transfection down-regulated SAV1 mRNA and protein levels in KGN cells, and the additional si-FMR6 reversed the down-regulation of SAV1 induced by si-SAV1. Then, CCK-8 analysis presented that si-SAV1 prominently restrained KGN cells proliferation compared with si-NC. Meanwhile, additional transfection of si-FMR6 could counteract the suppressor effect of si-SAV1 on cell proliferation (Fig. 4C). The results of Fig. 4D-E demonstrated that SAV1 knockdown promoted caspase-3 activity and apoptosis rate of KGN cells. However, downregulation of lncRNA-FMR6 eliminated this effect to a certain extent. In addition, the RT-qPCR experiments revealed that si-SAV1 enhanced Bax and reduced Bcl-2 expression in relative to si-NC group. Likewise, co-knockdown of lncRNA-FMR6 restored the effect of si-SAV1 on Bax and Bcl-2 expression (Fig. 4F). The above results indicated that SAV1 low expression promoted apoptosis and inhibited proliferation of KGN cells, as well as inverted the effect of lncRNA-FMR6 knockdown on KGN cells.

## Discussion

POF causes serious health damage to patients, including autoimmune diseases, osteoporosis, infertility, psychological distress, ischemic heart disease and increased risk of death [23]. Here, we reveal a novel mechanism of lncRNA-protein action that exerts a regulatory role in POF. It could be found that lncRNA-FMR6 was up-regulated in follicular fluid and OGCs of POF patients, low-expressed lncRNA-FMR6 inhibited KGN cells apoptosis and promoted proliferation, while over-expressed lncRNA-FMR6 exerted the opposite impact on KGN cells. Based on the perspective of mechanism, lncRNA-FMR6 binds to SAV1 and inhibits the transcription and translation of SAV1.

The role of lncRNAs in POF biology has become an area of great interest. For example, TRERNA1 is downregulated in POF and inhibits apoptosis in the granulosa cell line COV434 [24]. Activation of lncRNA-Meg3 can accelerates the promotion of apoptosis and induces premature ovarian failure in mouse OGCs [25]. Increased expression of DLEU1 is observed in POF, and DLEU1 can promote KGN apoptosis [14]. LncRNA-FMR6 is a novel lncRNA reported by Pastori et al. [26]. This lncRNA is an antisense transcript overlapping the 3’region of a microsatellite locus — FMR1 gene [26]. The current evidence suggests that lncRNA-FMR6 expression is downregulated in brain tissue of both Fragile X patients and mutation pre-carriers [26]. In addition, lncRNA-FMR6 accumulates in GCs of fragile X-associated premature ovarian failure [16]. Similar to the previous reports, in our study, we discovered that lncRNA-FMR6 was significantly overexpressed in follicular fluid and OGCs of POF patients. Besides, lncRNA-FMR6 was mainly distributed in the cytoplasm of KGN cells, conforming to the nature of lncRNA to exert its biological function after transcription [27]. Moreover, we also found for the first time that lncRNA-FMR6 inhibited KGN cells proliferation and promoted apoptosis. These findings suggest that lncRNA-FMR6 may come into play in facilitating POF progression.

LncRNAs participate in an extensive range of biological functions through various molecular mechanisms, including interactions with one or more protein partners in order to regulate the expression of cis-genes [21]. This regulatory network of lnRNA-protein has been found in the POF. For example, Zhao et al. [12] discovered that HOTAI was upregulated in ovarian tissues and serum samples from POF patients and inhibited hamster ovary apoptosis by upregulating protein expression of Notch-1. Li et al. [13] indicated that NEAT1 inhibited Chinese hamster ovarian cell lines Lec8 and CHO by suppressing p53 levels, which could thus alleviate POF. Wang et al. [28] considered that lncRNA HCP5 regulates DNA damage repair and GCs dysfunction by directly binding to YB1 and regulating its subcellular localization. Similarly, the present study identified SAV1, which is downregulated by lncRNA-FMR6 in POF. SAV1, located on chromosome 14q22.1, is a scaffolding protein regulating survival and apoptosis in a variety of cells [17, 18]. In this study, we found for the first time that SAV1 expression was down-regulated in POF and its low expression promoted cell proliferation and inhibited apoptosis of KGN cells, exhibiting the alleviating effect of SAV1 on POF. In addition, both RNA pull-down and RIP reflected the binding of lncRNA-FMR6 to SAV1. Pearson analysis revealed that an upscaling of SAV1 in POF was negatively correlated with lncRNA-FMR6. Combined with the negative regulation of lncRNA-FMR6 on the expression of SAV1 protein and the reverse effect of knock-down SAV1 on the biological function of KGN cells, we suggest that the promotion of POF of by lncRNA-FMR6 can be achieved based on the inhibition of SAV1.

SAV1 is a core kinase component of the Hippo signaling pathway and exerts an extensive and prominent role in regulating a variety of human cell biological behaviors [17, 18]. Therefore, in the future, we will concentrate on the impact of lncRNA-FMR6-SAV1 regulating Hippo pathway on POF. Moreover, in vivo experiments are essential to further verify the impacts of lncRNA-FMR6 and SAV1 on POF development.

## Conclusion

In general, lncRNA-FMR6 is overexpressed in POF, promotes apoptosis of KGN cells and inhibits proliferation by suppressing the expression of SAV1. Moreover, our study suggested that lncRNA-FMR6 and SAV1 might serve as a potential target for POF.

## Data Availability

Not applicable.

## Abbreviations

POF: Premature ovarian failure
SAV1: Salvador family WW domain containing protein 1
OGCs: Ovarian granulosa cells
lncRNAs: Long non-coding RNAs
RIP: RNA immunoprecipitation
